# Effects of EMG-Controlled Video Games on the Upper Limb Functionality in Patients with Multiple Sclerosis: A Feasibility Study and Development Description

**DOI:** 10.1155/2022/3735979

**Published:** 2022-04-11

**Authors:** Edwin Daniel Oña, Selena Marcos-Antón, Dorin-Sabin Copaci, Janeth Arias, Roberto Cano-de-la-Cuerda, Alberto Jardón

**Affiliations:** ^1^Robotics Lab, Department of Systems Engineering and Automation, University Carlos III of Madrid, Leganés 28911, Spain; ^2^Department of Physical Therapy, Occupational Therapy, Rehabilitation and Physical Medicine, Rey Juan Carlos University, Alcorcón 28922, Spain; ^3^International Doctorate School, Rey Juan Carlos University, Madrid 28008, Spain

## Abstract

Multiple sclerosis (MS) is the most common inflammatory neurological disease in young adults, with a high prevalence worldwide (2.8 million people). To aid in the MS treatment, using VR tools in cognitive and motor rehabilitation of such disease has been growing progressively in the last years. However, the role of VR as a rehabilitative tool in MS treatment is still under debate. This paper explores the effects of VR training using EMG activation in upper limb functionality. An experimental training protocol using video games controlled using an MYO armband sensor was conducted in a sample of patients with MS. Results support the use of EMG-commanded video games as a rehabilitative tool in patients with MS, obtaining favorable outcomes related to upper limb functionality and satisfaction.

## 1. Introduction

Multiple sclerosis (MS) is a chronic immune-mediated inflammatory demyelinating illness of the central nervous system of unknown etiology and multifactorial origin [1, 2]. MS is the most common cause of disability in younger adults. Among these disabilities, dexterity and activities of daily living (ADL) limitations on the upper limb (UL) represent one of the most common problems in patients with MS, usually affected by weakness or ataxia. Johansson et al. [3] reported that up to 76% of patients presented with any type of incapacity had UL impairment, and in at least 50% of patients, the severity of the dysfunction was moderate. Kamm et al. [4] and Choi et al. [5] reported that, after 15 years of disease evolution, the majority of MS patients report hand's problems at the functional level, so patients perform compensations or decrease UL functions [4, 5], although its importance may be underrecognized relative to walking impairment, which is the hallmark symptom of MS [6].

Neurorehabilitation for UL in people with MS is aimed at maintaining or improving function and quality of life [7]. It can be strengthened with technology, such as robotics, virtual reality, or functional electrical stimulation, to introduce the key elements of motor learning (feedback, intensity, error-based learning, practice of functional tasks, repetition, motivation, and attention) [8, 9]. However, the costs of these devices are one of their main limitations, so new research is needed to improve manual dexterity in people with MS using technology, adding adherence and motivation to this chronic illness.

In this paper, the feasibility of EMG-controlled video games as a rehabilitation tool in MS treatment is studied. For that purpose, a pilot study was conducted at a multiple sclerosis center with seven patients using a series of video games commanded by the 8 EMG channels' low-cost armband sensor, such as MYO sensor, during a training protocol defined by therapists.

## 2. Related Work

Currently, techniques based on electromyography (EMG) reveal a potential clinical value in healthcare with a specific focus on neurorehabilitation [10, 11]. The EMG signals are formed by the variations in states of skeletal muscles, which are triggered by the human intention transferred from neurons to corresponding limbs. These signals can be acquired by invasive or noninvasive methods. Although the invasive methods provide better signal quality and more precision, this approach involves surgical procedures for electrode implantation, which present some inconveniences such as the risk of infectiousness, contingent rejection reaction, discomfort, and other medical concerns.

On the other hand, the noninvasive approaches present a lower quality of signal but are easy to use, and in commercial terms, there exist different models depending on the final applications. Devices capable of acquiring the superficial electromyography (sEMG) signals can be commercially founded in different forms, such as bands for measuring the muscle activity in the arms or devices with separate electrodes, with different communications protocols such as wireless, Bluetooth, or cable and with different quality of signals depending on the low-level filtering and the electrode material quality. All these characteristics influence the price of the device.

Different factors need to be considered in the sEMG signals acquisition, for example, the skin factors such as the sweat or the hair, the electrode location over the muscle, the muscle fatigue, different noises produced by the muscles moving, and the harmonics induced by the power line 50 Hz or 60 Hz. Also, the signal amplitude is of the order of mV, and it needs to be amplified before its use.

Usually, three steps of signal preprocessing are applied before using the signal in different applications. This preprocessing consists of rectification, filtering, and signal normalization. The sEMG signal presents *x*-axis symmetry, which makes its average value have low and insignificant values. In this case, it is a good practice to rectify sEMG a priori, considering only its absolute value. This rectification doubles the sEMG signal frequency. Signal filtering is necessary for noise reduction. In general, a 60 Hz band-pass filter (50 Hz in Europe) is used to remove noise characteristics of the power line and with lower cutoff frequencies between 5 and 20 Hz to remove noise variation from the zero line or motion artifacts. Signal normalization is an option to prevent noise variation, and it does not influence the classification process. In this work, only the default MYO armband filters are used. No additional filter and normalization were implemented.

The built-in proprietary system of the MYO armband is limited to the recognition of 5 gestures. Moreover, when the MYO armband is used in a user-independent scenario, which means that it can be used by new users without prior training, its recognition accuracy drops from 83.1% (user-dependent) to 53.7% (user-independent) [12]. Thus, customized developments have been conducted in several fields.

MYO can communicate on real-time hand gesture recognition using deep learning techniques with PCs via Bluetooth, virtual environments, or other objectives such as prosthesis [13], a steering assistance interface [14], an augmented reality dance game designed to improve rehabilitation therapies in upper limb amputees while the hand gestures are analyzed using EMG data collected by MYO [15], or an EMG intention detection system based on the MYO armband to control robotic hand orthosis [16]. It has been recognized as a noninvasive, more user-friendly, and time-saving device compared with conventional electrodes [17].

To the best of our knowledge, very few studies have been conducted with MYO armbands to treat dexterity dysfunctions in people with neurological disorders and none with people with MS for treatment aims. Macintosh et al. [18] recruited 19 people with cerebral palsy during a 4-week home-based intervention with movement-controlled video games with MYO. Lyu et al. [19] tested MYO in 2 patients with chronic stroke to distinguish between the desired movement strategy and unwanted alternatives, previously tested in six healthy participants that practiced the task for one session with a total of 144 trials. Sadeghi Esfahlani et al. [20] determined accurately the range of motion and the kinematic ability combining Kinect technology, MYO sensor, and a FootPedal with a semi-immersive virtual environment called ReHabGame in 10 healthy participants and 2 poststroke patients, 2 traumatic brain injury patients, and 9 with multiple sclerosis, as an inexpensive and home-based assessment tool through of serious game that comprised four scenarios. Finally, Totty and Wade [21] performed a study with 10 nondisabled individuals to remote monitoring the physical activity using the MYO sensor in order to assess the feasibility of classifying categories of activities of daily living from the Functional Arm Activity Behavioral Observation System (FAABOS) scale using muscle activation and motion data.

The aim of this feasibility study was to evaluate the effects of EMG-controlled video game-based training, combined with conventional rehabilitation, for improving the UL functionality (grip muscle strength, coordination, dexterity, fatigue, and quality of life) in patients with MS. Furthermore, a secondary goal is to assess satisfaction, adherence, presence of cybersickness, and workload perceived by participants on treatment.

## 3. Materials and Methods

**Figure 1:**
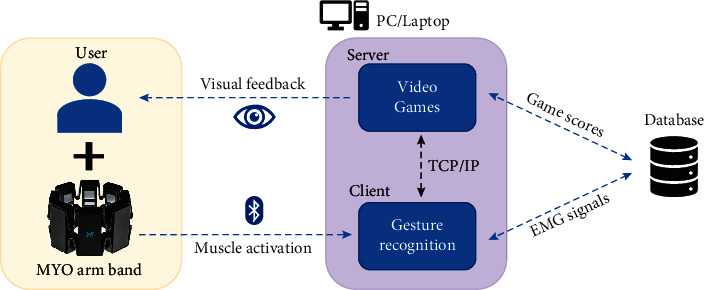
Proposed framework for EMG-based upper limb rehabilitation.

This paper studies the effects in UL functionality of a training protocol based on conventional rehabilitation plus an experimental method using gaming technology. The experimental VR-based method consists in promoting the arm muscle activation by commanding the video game actions with EMG sensors. The system used as a rehabilitation tool is made up of two subsystems: (1) the set of video games and (2) the gesture recognition system. Both subsystems were implemented in different development platforms due to their particular characteristics of them. The results of the gesture recognition block are sent to the video game through TCP/IP protocol, being the video game on the server-side and the gesture classifier on the client side.  Figure 1 depicts the components of the proposed system and describes the minimum infrastructure employed in this study.

On the one hand, the serious games (SG) implemented for this study aim to imitate movements included in conventional physical therapy, such as pronation, supination, grasping, and wrist deviations, but adding the motivational effects of gaming technology. This rehabilitation strategy using SG is proposed for patients with MS but can be used by people with limited mobility in order to restore their ability to independently perform the activities of daily living (ADL) or to recover a lost or diminished function. The user can interact with the video game using arm gestures, which are detected by EMG sensors. Thus, when the user performs a hand gesture, an action is commanded in the video game.

On the other hand, the gesture recognition system is based on a neural network classifier that identifies the EMG muscle activation when a gesture is performed. The muscle activation is detected by an MYO sensor located in the user's forearm, and a flag bit is sent to the video game when a gesture is successfully identified. Thus, the user can interact with the video game naturally since the sensor does not interfere when gesture performing.

As illustrated in Figure 1, both the video game and the gesture recognition block are executed on a single PC or laptop; however, they require different SW conditions. The video games are installed in the operating system, so they work in a standalone manner. The gesture recognition system requires its native development software to be running. Consequently, a method for information transferring is needed. In this case, a server-client method was implemented to communicate the video games and the gesture recognition block.

Additionally, two data types are gathered automatically during the video game performing: (1) the video game scoring and game settings and (2) the raw data of EMG muscle activation. This information can be used in further analysis to better understand the patient's performance and the relationship with the game conditions (difficulty, type, repetitions, etc.).

### 3.1. Development Tools

#### 3.1.1. Hardware Tools

The MYO armband (Thalmic Labs, Kitchener, ON, Canada) sensor was used to detect forearm muscle activity. This sensor is a gesture recognition band that could work without extra batteries and transfer data wirelessly with adequate sample frequency and accuracy [22]. Specifically, the MYO armband is comprised of eight dry sEMG sensors and one 9 degrees of freedom (DOF) IMU. Each EMG sensor is sampled at a frequency of 200 Hz and outputs an eight-bit unitless integer value that ranges from −128 to 127 representing the level of activation of the muscle being sensed. The 9 DOF IMU contains a three-axis accelerometer, a three-axis gyroscope, and a three-axis magnetometer, each one sampled at a frequency of 50 Hz.

#### 3.1.2. Software Tools

The video games were developed using the Unity 3D game engine and C# programming for the game scripts. This open-source engine allows the video games created to be accessible and free. Additionally, the MATLAB software was used to implement the gesture recognition system based on the Neural Network Toolbox. The MYO Connect application was used to capture and send the EMG signals to MATLAB software. The MYO Connect application receives via Bluetooth the raw data sent by the MYO armband.

## 4. System Description

As previously mentioned, the proposed system consists of two main subcomponents: (1) the set of video games commanded by EMG activation and (2) the gesture recognition system that identifies the movements performed by the user's arm. The two subcomponents are described in deep as follows.

### 4.1. Video Games for the MYO Sensor

**Figure 2:**
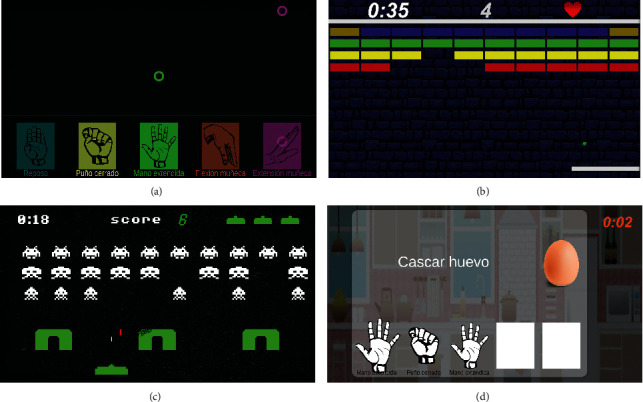
Proposed framework for EMG-based upper limb rehabilitation. (a) MYO-Gesture. (b) MYO-Arkanoid. (c) MYO-Space Invaders. (d) MYO-Cooking.

A total of four video games were implemented for this pilot trial (Figure 2), namely, the MYO-Gesture, the MYO-Arkanoid, the MYO-Space Invaders, and the MYO-Cooking. A set of eight hand gestures are used to command the actions in the games; six of them are detected by the MYO armband based on the EMG activation. The remaining two gestures (pronation and supination movements) are detected using the inertial measurement unit (IMU) of the MYO sensor.

The video games share several functions like patient record management, gestures mapping, secure login, automatic data storage, and feedback to the patient. However, the gameplay and particular functionalities of each game are described in detail as follows.

#### 4.1.1. MYO-Gesture Game

The MYO-Gesture game (see Figure 2(a)) imitates the mechanics of the well-known ”Guitar Hero” video game. In this case, a series of colored rings are sequentially falling in different columns along the screen width. Each column is assigned to a specific hand gesture, and the player must imitate the gesture shown in the column in which the ring falls. There are five positions/columns implemented for training five gestures at a time and in order to do not reduce the icon's size. However, the therapist can choose whether or not all the gestures will be used. Prior to starting the game, the therapist can choose from one to five gestures to be used in the round game in order to intensify the activation of a specific muscle group.

Similar to the Guitar Hero game, the goal of this game is to complete the music by properly replicating the hand gestures required by each ring. When the player imitates correctly the gesture required, a point is given, and a part of the music is reproduced. Contrary, when the gesture required is not performed, a gap in the music is obtained, and the music will be incomplete. Thus, the purpose of the audio feedback is to provide the user with an immediate quantitative evaluation of how well they performed the gestures during gameplay.

This video game offers several options to customize the difficulty level according to the patient needs, such as the game duration time, the game style (autocompleted or playback soundtrack), the ring spawn time, the ring falling speed, the music theme, and gesture mapping. This last option allows for choosing the hand gestures to be trained. It is because only five gestures can be displayed on the screen, but a set of eight gestures is available.

#### 4.1.2. MYO-Arkanoid

The MYO-Arkanoid game (see Figure 2(b)) implements the mechanics of the arcade game with the same name. In this game, the user takes control of a paddle at the bottom of the screen and must use it to deflect a ball into rows of blocks at the top of the screen, thus destroying them and eventually clearing the screen. Each block destroyed by the ball is equivalent to one or five points if it is a golden block. The game ends when the player destroys all the blocks or loses all lives. By default, the player has three lives, and it loses one when the ball falls at the bottom of the screen.

For this game, the following options were included to personalize the difficulty level: short or large paddle size, game mode (a single or three balls simultaneously), paddle displacement speed, ball speed, and gesture mapping. This last allows for choosing the hand gestures to command the paddle motion. Due to the paddle can move horizontally, only two hand gestures can be used in this game.

#### 4.1.3. MYO-Space Invaders

This is a fixed shooter in which the player moves a laser cannon horizontally across the bottom of the screen and fires at aliens overhead. The aliens begin as three rows of ten that move left and right as a group, shifting downward each time they reach a screen edge (see Figure 2(c)). The aliens attempt to destroy the player's cannon by firing projectiles. The game mechanics lies in dodging the alien attacks by using two gestures to move the spaceship laterally and to destroy all of the aliens by shooting them using a third gesture. Thus, three gestures can be used in this video game.

The player's cannon is partially protected by stationary defense bunkers, which are gradually destroyed from the top by the aliens and, if the player fires when beneath one, the bottom.

For the MYO-Space Invaders, the following options were included to personalize the difficulty level: number of lives, rate of alien attacks, firing speed, spaceship speed, spaceship tracking (focusing the aliens' attacks on the spaceship), and gesture mapping. For this game, this option allows choosing the hand gestures to command the spaceship motion and laser firing. Consequently, only three hand gestures can be used in this game.

#### 4.1.4. MYO-Cooking

In this game, players get to be the chef, and they have to prepare a dish by following the steps included in a recipe (see Figure 2(d)). Firstly, the therapist has to create a recipe indicating the ingredients and steps to prepare the dish. A recipe is composed of several steps with different ingredients. In each step to create the dish, the therapist must associate various hand gestures with a particular ingredient. Each dish step involves a sequence of hand gestures. For example, Figure 2(d) illustrates a sequence of hand opening, grasping, and hand opening again to crack the egg.

Thus, the gameplay is to imitate the gesture sequence shown on the screen to complete the proposed recipe step. The recipe step is completed when the player performs all the gestures associated with it, and the game ends when the player fulfills all the recipe steps. The maximum number of gestures associated with a recipe step is five.

The customization options of MYO-Cooking are different from previously described games since it requires the recipe's creation by the therapist prior to intervention. The video game offers a friendly menu-based interface to create recipes, add a new ingredient, and edit or import a recipe. Additionally, in each recipe step, the therapist can include a description of the task to help the patient understand the required task. In order to create a recipe, the therapist can expend various minutes depending on the recipe extension (number of recipe steps, number of gestures used, description of steps, etc.).

### 4.2. Gesture Recognition System

**Figure 3:**
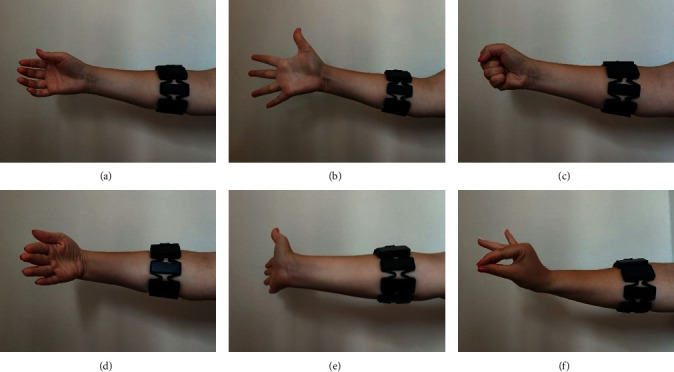
Set of gestures identified for the sEMG recognition system. (a) Relaxed hand. (b) Extended hand. (c) Handgrip. (d) Wrist flexion. (e) Wrist extension. (f) Pinching.

The MYO armband is provided with a real-time hand gesture recognition algorithm for fist (hand closed), wave in (wrist flexion), wave out (wrist extension), and fingers spread (hand open). Also, the double-tap (accomplished by tapping the thumb to the forefingers twice in quick succession) is used to lock/unlock the device. In this work, according to the rehabilitation therapy, the developed application needs to identify the following gestures: relax, grip, extended, wrist flexion, wrist extension, and pinching (see Figure 3), to which the movement of pronation and supination from the IMU signals is added.

**Figure 4:**
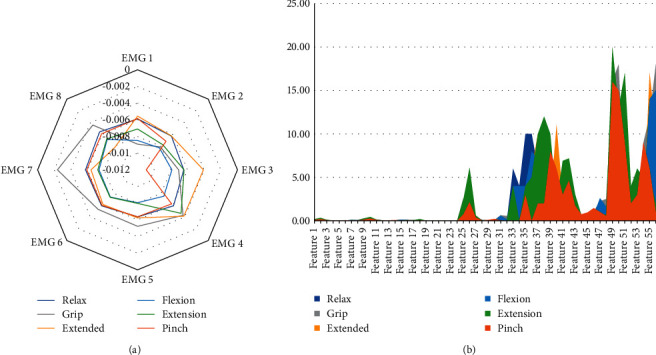
EMG signal levels for different gestures. (a) The amplitude of the EMG signals for different gestures. (b) The features amplitude for different gestures.

The developed application uses the sEMG signals provided by the 8 MYO armband sensors, rectified, filtered, and normalized. Only the rectification process was implemented in this work because the signals were filtered and normalized by the Myo SDK [23]. An example of these sEMG signals levels according to the proposed gesture recognition is presented in Figure 4(a). This represents an average level of 1000 samples for each of the eight electrodes for each proposed gesture. After sEMG acquisition, the adjacent segmentation technique was used, where the sEMG signals are split into adjacent windows. According to Oskoei and Hu [24], a real-time classification is considered when the length of the segment does not exceed 300 ms, but the longer the segment, the more accurate the gesture classification. For this reason, segments were fragmented into windows with a fixed length of 300 ms. In each window, time-domain features were calculated: (1) mean average value (MAV), (2) root mean square (RMS), (3) variance (VAR), (4) signal strength indicator (SSI), (5) zero crossings (ZC), (6) wavelet transform (WL), and (7) side scatter (SSC). In total, 56 values that represent the 7 features are extracted from each window, that is, from 1 to 8, the first feature, mean average value for each electrode (8 electrodes), from 9 to 16, the second feature, root means square for each electrode, and so on. These characteristics for each proposed gesture recognition can be seen in Figure 4(b). Although some features have very low values (close to 0), they have a significant influence on the classification of gestures.

The time-domain features used in this work are detailed as follows:

(i) *MAV*. Detect the muscle contraction levels. It is calculated by taking the mean of the absolute value of the signal *x*_*i*_ in the segment *i* that is *N* samples long and is expressed by (1) [25]:
  (1) $$\begin{array}{c} MAV_{i}=\frac{1}{N}\overset{N}{\underset{k=1}{\sum }}\left|x_{k}\right|,\;\text{for }i=1,2,\ldots ,I, \end{array}$$
where *x*_*k*_ is the *k* th sample of the segment *i* and *I* is the number of segments.
(ii) *RMS*. It is related to the constant force and nonfatigued contraction of the muscle. It refers to the standard deviation, and it is expressed by (2) [24]:
  (2) $$\begin{array}{c} RMS=\sqrt{\frac{1}{N}\overset{N}{\underset{i=1}{\sum }}x_{i}^{2}}. \end{array}$$
(iii) *VAR*. Starting in the late 1970s, the EMG signal was modeled as amplitude-modulated Gaussian noise whose variance is related to the force developed by the muscle [26, 27]. The variance (or second-order moment) of the EMG signal forms another parameter related to the power of the EMG signal. The variance is classically defined as an average of the deviation of the signal from the mean at each point. As the EMG signal usually has a mean very close to 0, its variance is usually expressed as
  (3) $$\begin{array}{c} \text{VAR}=\frac{1}{N-1}\overset{N}{\underset{i=1}{\sum }}{\left(x_{i}-M\right)}^{2}, \end{array}$$
where *M* is the mean value of the EMG signal [28].
(iv) *SSI*. SSI forms an index on the energy of the signal. It is defined mathematically as [29]
  (4) $$\begin{array}{c} SSI=\overset{N}{\underset{i=1}{\sum }}x_{i}^{2}. \end{array}$$
(v) *ZC*. It is the number of times that the signal passes through zero or the number of times that the signal changes the sign in a given segment [30]. It provides an estimate of the frequency. An amplitude threshold must be included to avoid zero crossings produced by signal noise. ZC increment the count, if *x*_*k*_ > 0 and *x*_*k*+1_ < 0 or *x*_*k*_ < 0 and *x*_*k*+1_ > 0 and *|x*_*k*_ − *x*_*k*+1_*|* ≥ *threshold* [25].
The threshold is included to reduce noise, and *x*_*k*_ and *x*_*k*+1_ are consecutive samples. This parameter provides a rough estimate of the frequency domain properties.
(vi) *WL*. WL is the cumulative length of the waveform over the time segment.
  (5) $$\begin{array}{c} l_{0}=\overset{N}{\underset{n=1}{\sum }}\Delta \left|X_{n}\right|, \end{array}$$
where Δ*X*_*n*_=*X*_*n*_ − *X*_*n*−1_, the difference between two consecutive samples. The resulting values give a measure of the amplitude, frequency, and duration of the signal shape, all within a single parameter [25].
(vii) *SSC*. It is the sign change of the slope of the signal and provides a measure of the frequency of the measured signal. Given three consecutive samples *X*_*n*−1_, *X*_*n*_, and *X*_*n*+1_, the sign change of the slope of the signal will be increased if
  (6) $$\begin{array}{c} SSC=\overset{N}{\underset{i=3}{\sum }}\mathrm{sgn}\left[-\left(-x_{i}-x_{i-1}\right)\left(x_{i-1}-x_{i-2}\right)\right., \end{array}$$
where sgn(*x*)=1 when *x* > 0 and 0 otherwise.

The all process of the gesture classification implemented in the application consists of three steps: data acquisition, neural network training, and validation. After validation, the gesture recognition application can connect to Unity to start a new game. Data acquisition consists in creating a new dataset with 100 samples (sEMG features) for each gesture, in total 600 samples. After the MYO armband was positioned over the forearm and was synchronized with the computer, the user is asked to imitate the six gestures during which the acquisition of sEMG signals is made, and the characteristics are extracted. The features are stored in the dataset with the name of each gesture.

**Figure 5:**
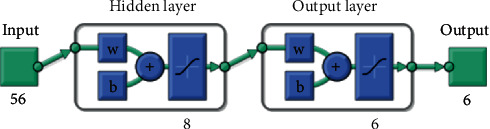
Architecture of neural networks.

The second step consists in processing the data stored in the dataset to be used in neural network training. This is a predefined feedforward neural network with 56 inputs (the sEMG features), 2 layers of 8 and 6 neurons with a log-sigmoid activation function. The output of the neural network is represented by a 6-position vector, where each position represents a gesture recognition with a value between 0 and 1: 0 indicates that the gesture is not recognized, and 1 indicates that the gesture is recognized 100%. The neural network architecture used in this work can be seen in Figure 5.

**Figure 6:**
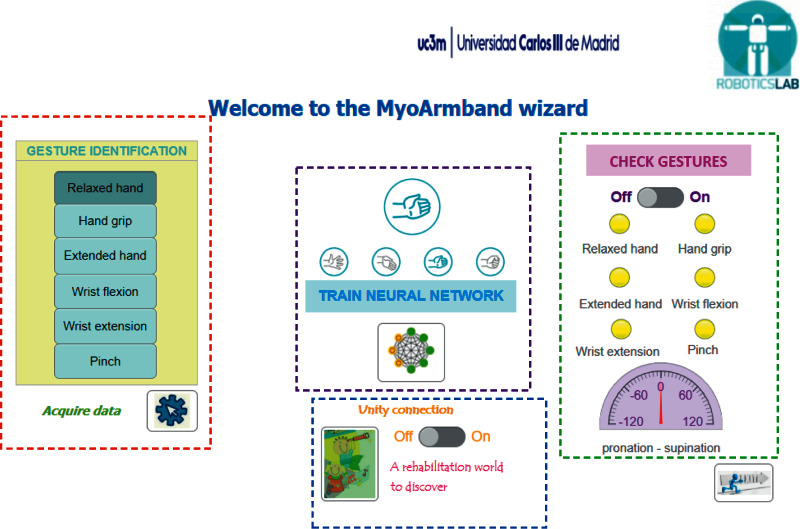
GUI for system's connection.

The proposed interface to automate the process of data acquisition, NN training, gesture verification, and connection to the Unity video games can be seen in Figure 6. The interface permits creating a personalized dataset with predefined gestures for each user and training the NN architecture. Compared to the literature, the proposed method is to personalize the dataset according to the patient and do not use a dataset that contains samples from different subjects. With this point of view, a personalized dataset offers more accuracy but increments the setup time necessary for data acquisition and NN training.

The right side buttons from the interface (framed in red dotted line in Figure 6) represent the predefined gestures and are used for data acquisition. The user is asked to replicate the selected gesture (the gestures will be selected one by one), and the application automatically stores 100 samples with the name of this gesture in the dataset. Once the dataset was created with all the gestures (600 samples), the button located in the central zone (framed in mauve dotted line) permitted the NN training. A script automatically concatenates all samples, generating two matrices with the input and output data (target data) for the supervised learning algorithm, with which the NN is trained. All processes from the data acquisition to the validation for a new user, depending on the user and the computer power, may take between 3 and 5 minutes. The validation process (the framed in green dotted line from Figure 6) can be activated with a switch button and consists of lighting the LEDs depending on the detected gesture: yellow light if the validation process is off, green if the gesture has been recognized, and red if the gesture has not been recognized.

At the bottom of the application, the pronation-supination angle coming from the IMU sensor is represented by a semicircular gauge between −120 and 120 degrees. Also, during the validation process, the NN output is stored in a column vector of six positions with values between 0 and 1, where the maximum value represents the recognized gesture. This vector is increased with three positions, representing the normalized Tait-Bryan angles (yaw, pitch, and roll), and is sent to the Unity program to manage the game movements. These angles are obtained from the rotation matrix provided by the Myo SDK [23]. These are values between −180 and 180 degrees, and for their normalization, it was divided by 180, obtaining a value between −1 and 1. For the roll angle, from −1 to 0 represent the pronation movement and from 0 to 1 the supination movement. The control of the video game is according to threshold exceeding in order to include a dead zone for a central/neutral movement. For example, if the angle is less than −0.3, a pronation action is detected and if greater than 0.3 a supination action is identified. However, while the arm keeps between the range of −0.3 to 0.3, neither pronation nor supination actions are identified. This threshold can be adapted for changing the difficulty level in the game.

#### 4.2.1. Neural Network Performances

**Figure 7:**
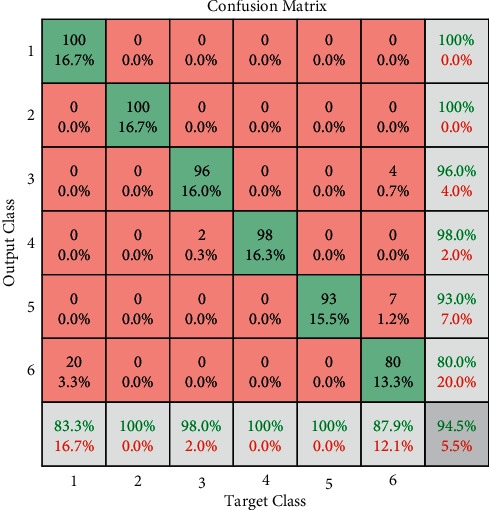
Confusion matrix for gesture recognition.

A new dataset with 600 samples was stored for a new user. This dataset contains 100 samples of each movement, where in each sample, a vector of 56 positions was saved, which represent the 7 characteristics. These characteristics were extracted from the MYO armband sEMG signal (8 electrodes) during each 300 ms. For the neural network training, the whole dataset was divided randomly into 70%, 15%, and 15%. 70% of data were used for training, 15% were used for validation, and another 15% were used for network testing. The training algorithm was based on the Levenberg–Marquardt algorithm, and the performance was measured with the Mean Squared Error (MSE). After network training and its validation with the aid of the application, a new dataset was stored with the same gestures, but in this case, at the same time, the response of the trained neural network. These new values from the NN output were used for evaluating the NN performance. The target data were plotted according to the NN output in the confusion matrix (see Figure 7), where the rows correspond to the predicted class (output class, gesture recognized by the NN), and the columns correspond to the true class (target class, gesture made by the user).

In Figure 7, the numbers from 1 to 6 represent the 6 gestures, and the diagonal cells (in green) correspond to observations/gestures that are correctly classified. The off-diagonal cells correspond to incorrectly classified observations. In this example, as can be observed, gesture recognition is 94.5% accurate. The predicted percentage of each gesture is presented in the last row, and the columns correspond to the true class (target class). The weakest predictions (last row) are in the relax gesture (83.3% accuracy) and pinch gesture (87.9% accuracy). The 83.3% of accuracy for the relax gesture is due to the fact that 20 samples of this class were classified in the relax class. It should be noted that the pinch gesture was stored by not exerting too much force between the thumb and index finger; otherwise, confusion will occur between the extension and pinch gesture. Also, the 87.9% of accuracy for the pinch gesture prediction is influenced by the 7 samples from the wrist extension class and 4 samples from the extended hand class, wrong classified in the pinch class. The last column represents the percentage of the true class, where, for example, 80% of the samples from the pinch gesture class were well classified, similarly, 93% for the wrist extension, 98% for the wrist flexion, 96% for the extended hand, and 100% for the handgrip and relax gesture.

### 4.3. System Connection

In Figure 6, the switch button “Unity connection” (framed in blue dotted line zone) permits connecting the gesture recognition application with the Unity video games when the verification button also is on. The data are sent by TCP/IP protocol, where the gesture application is a client who connects with the server, Unity video games. The client sends to the server the gestures vector of 9 positions representing the 6 gestures and the 3 normalized Tait-Bryan angles.

## 5. Feasibility Study

A case series study was conducted following the CARE report guidelines [31] as a feasibility study. Nonprobabilistic sampling of consecutive cases was used. All interventions were performed at the Leganés Association of Multiple Sclerosis (ALEM) in Madrid, Spain. Informed consent was obtained from all participants included in this study. This research was approved by a local ethical committee (reference 26/12).

### 5.1. Participants

The initial sample consisted of 9 patients. However, 2 of them were excluded because they could not attend the association twice per week to receive treatment. Thus, the final sample consisted of 7 patients (6 men and 1 woman), 3 of them with secondary progressive MS, 3 with Relapsing-remitting MS, and 1 patient with primary progressive MS. Four patients had greater involvement on the left side and three on the right side. The age of the patients ranged between 29 and 56 years (mean 46.57 ± 9.71 years). Regarding the EDSS scale score, the sample ranged between 3.0 and 7.0 (5.43 ± 1.43). The mean time of evolution of the disease was 14.43 ± 9.5 years. All descriptive sociodemographic data are shown in Table 1.

All patients fulfilled these inclusion criteria: a confirmed diagnosis of MS between 3.0 and 7.0 on the Kurtzke Expanded Disability Status Scale (EDSS); stable medical treatment during at least the six months prior to the experimental intervention; modified Ashworth Scale ≤2 points in the upper limbs; a score ≥24 points in the Mini-Mental Test.

The exclusion criteria were a diagnosis of other neurological illnesses different to MS; suffering an outbreak in the prior three months to the present research; having received a cycle of steroids six months prior to the experimental protocol and during the study; having received treatment with botulinum toxin in the six months prior to the beginning of the research; perceptual and visual disorders no corrected by optical devices.

### 5.2. Intervention

All patients received conventional rehabilitation for the UL by 1 physical therapist between May and July of 2021. The conventional protocol was 45 minutes, 2 sessions per week, based on gross and fine motor coordination, mobilization, strengthening and stretching techniques, and practice of dexterity and daily living tasks based on prior studies [14, 15].

Additionally, an experimental protocol was conducted based on serious games designed for the MYO sensor. Experimental treatment was scheduled from 12 to 20 minutes per session, twice per week over an eight-week period (16 sessions for all patients). Each session was focused on one upper limb alternatively (more affected side one session and less affected side the next one).

### 5.3. Experimental Protocol

The video games attempt to exercise a full range of motion in the UL, as well as promote repetitive movements in a friendly manner. The movements involved are relaxed hand, extended hand, handgrip, wrist flexion, wrist extension, pinch, forearm pronation, and supination.

Prior to performing a training session, the set of gestures must be calibrated for the particular conditions of each participant. The calibration process uses MATLAB software to feed sEMG data from the MYO armband sensor (placed on the forearm) wirelessly into the neural network classifier. Every time the MYO is switched on, a new movement calibration must be performed to ensure correct hand gesture detection. The process takes approximately 4-5 minutes overall, and it has to be done every time a new user interacts with the system. After that calibration, the participant can command the video games using the EMG generated when a gesture is performed.

The experimental protocol consisted of the application of the four video games, increasing the game intensity and modifying the gesture mapping on each game. Gesture mapping of each game was assigned by the therapist according to the treatment evolution. Thus, the protocol programmed for the participants in this study is shown in Table 2. Note that the experimental protocol was performed with the more and less affected arm at each therapy session.

### 5.4. Outcome Measures

In order to evaluate the effectiveness of treatment, three assessment stages were conducted: previous to intervention (baseline), at the end of treatment (final), and two weeks after treatment (follow-up). All assessments were performed by the same two raters trained in the use of the measures, blinded to the interventions. The following outcome measures were used.

A hydraulic hand dynamometer (Jamar®) was used to assess grip strength. All the patients performed three grip movements, and the mean values were recorded. Hand dynamometry is recommended by the American Society of Hand Therapists and by the Brazilian Society of Hand Therapists [16]. The data were recorded in kilograms for both sides.

The Box and Block Test (BBT) was performed to measure coordination, speed of movements, and gross dexterity for both sides. Patients are instructed to move as fast as possible the maximum number of blocks from one side to another side of a box within one minute. The BBT is a quick, simple, and reliable assessment. Its administration and its validity have been shown in subjects with upper limb disability [17, 18].

Nine Hole Peg Test (NHPT) was used. It is a hand function test, which consists of a plastic pegboard with nine holes and nine pegs. The patient is instructed to put the nine pegs in the pegboard as fast as possible and then remove them again for both sides. The time is recorded as an outcome measure in seconds (seg.) [19].

The ABILHAND is a measure of 23 bimanual activities for adults with upper limb impairments [32]. The scale measures a person's ability to manage daily activities that require the use of the upper limbs, whatever the strategies involved. The questionnaire is downloaded from the website and one of the 10 random orders of questions. These are read to the patient and scored as either “impossible,” “difficult,” or “easy.” If a task has not been attempted in the last 3 months, then it is marked as N/A.

The Fatigue Severity Scale (FSS) is a 9-item scale, which measures the severity of fatigue and its effect on a person's activities and lifestyle in patients with a variety of disorders [33]. It was originally devised for people with multiple sclerosis or systemic lupus erythematosus. The subject is asked to read each statement and circle a number from 1 to 7, depending on how appropriate they felt the statement applied to them over the preceding week. A low value indicates that the statement is not very appropriate, whereas a high value indicates agreement.

Quality of life was assessed by the Multiple Sclerosis Impact Scale (MSIS-29). It presents two dimensions: physical and psychological well-being. It is conformed by 29 questions. Items are scored from 1 to 5, with 5 being a worse quality of life perceived. The maximum score on the physical part is 100 points and 45 points for the psychological well-being part [20, 21].

Satisfaction was assessed with the Client Satisfaction Questionnaire (CSQ-8) and a specific questionnaire that evaluated satisfaction with technology for all patients recruited. CSQ-8 consists of eight dimensions that assess the satisfaction with the care and treatment received. The total score is 32 points, with higher scores meaning higher satisfaction [34]. Satisfaction with technology and with the MYO treatment program was also assessed with a questionnaire previously designed and used by our research team [35]. The dimensions considered are technical quality and operation of the equipment; ease of the video game to be played; program compliance and satisfaction in relation to the treatment performed; general degree of satisfaction. Each dimension is scored from 1 to 5, with 5 being very satisfied. A total score is also calculated as a percentage (%).

The System Usability Scale (SUS) also was used. It is a reliable tool for measuring the usability and consists of a 10-item questionnaire with five response options for respondents, from strongly agree to strongly disagree [36]. Originally created by John Brooke in 1986, it allows you to evaluate a wide variety of products and services, including hardware, software, mobile devices, websites, and applications. The best way to interpret your results involves “normalizing” the scores to produce a percentile ranking.

The Short Symptoms Checklist (SSC) comprises symptoms (two taken from each of the SSQ subscales of nausea, oculomotor, and disorientation) [37]. Participants are asked to rate the severity of each symptom on a five-point scale (“not at all,” “slightly,” “moderately,” “definitely,” and “severely”) up to 45 minutes after immersion. Although it has not been validated yet as an independent measure, the SSQ provided convenient profiling of symptoms experienced during immersion.

NASA-Task Load Index (NASA) scale was used to assess perceived workload. It is divided into six parts (mental demand, physical demand, temporal demand, performance, effort, and frustration). Each part is analyzed in a percent value (%), and a total score is also calculated [38]. Additionally, we recorded the attendance rate (%) for therapy sessions (compliance).

### 5.5. Statistical Analysis

SPSS statistical software system (version 28.0) was used. A descriptive analysis was conducted. The Shapiro–Wilk test was used for normality analysis (*n* < 50). The use of nonparametric statistical tests was considered adequate because the data did not follow a normal distribution (*n* < 30). Friedman test for related samples was used to compare variables. Moreover, the Wilcoxon test for paired samples was used to compare variables throughout the measurements. A 95% confidence level was assumed. *P* values < 0.05 were considered significant. In addition, the correction of the type I error in the Wilcoxon test was taken into account.

## 6. Results

The efficacy of MYO-controlled video game-based training in MS treatment was estimated in terms of handgrip strength, both gross and fine manual dexterity, performance in the ADL, quality of life, and satisfaction. There was not a discontinuity in the patient's tracking (all the participants were assessed in all stages). The results obtained by the outcome measures are summarized as follows.

The scores of the Jamar handgrip dynamometer for each participant are shown in Table 3. The measurements yielded a result of clinical improvement on handgrip strength on both sides across the measurements, but no statistical significance was achieved.

Regarding the gross manual dexterity estimated by the BBT, a notable clinical improvement was observed in the measurements presented in Table 4. In addition, statistical significance was obtained in the Friedman test (more affected *p* = 0.042; less affected *p* = 0.034). For this reason, the Wilcoxon test was done, but in this case, no significance was obtained.

There were no clinical changes in fine manual dexterity throughout the initial measurement and measurement at the end of treatment for both sides, according to the NHPT measurements shown in Table 5. However, there was a slight worsening in the follow-up after treatment. No statistical significance was recorded throughout the measurements.

The data related to the FSS (52 points on average) and the ABILHAND (40 points on average) remained stable over time, without obtaining statistically significant changes in the paired comparisons. The results regarding the quality of life measured through the MSIS-29 showed a clinical worsening, which was greater in the physical dimension than in the cognitive one. Despite these data, no significant changes were recorded. A summary of the above scores and the statistical analysis are shown in Table 6, including the previous outcome measure results.

Satisfaction with technology and MYO treatment obtained an average score of 70.29 ± 7.13 out of 100 points (see Table 7). The medium CSQ-8 score was 80.35 ± 10.93 out of 100 points.

The mean score obtained in SUS was 74.64 ± 8.47 out of 100. The mean score relative to the SSQ scale on symptoms of distress was 11.22 ± 9.66 points and 21.62 ± 10.55 points at NASA-Task Load Index on a 100-point scale.

The percentage of registered attendance had an average of 92.93% ± 13.7 to the experimental protocol sessions proposed.

## 7. Discussion

Despite the fact that previous studies combining video games and EMG muscle activation have shown potential clinical benefits [39], EMG systems were not routinely utilized for intervention following neurologic injury. Limited time and resources were identified by clinicians as key barriers to implementing new clinical practices. Hence, new perspectives on the design of EMG-based system are needed for streamlined, intuitive, and clinically effective applications [39].

In this line, the goal of this study was to evaluate the effectiveness of a combined rehabilitation protocol focused on the UL in MS treatment. The rehabilitation protocol was based on conventional therapy plus an experimental protocol based on video games controlled by EMG activation. Results of the pilot trial indicate that sEMG-based video game treatment is feasible to improve the UL functional capacity in patients with MS but does not cover all the functional spectrum. Namely, gross manual dexterity and handgrip strength were improved according to the outcome measures of the BBT and Jamar dynamometer, respectively. However, fine manual dexterity measured by the NHPT presented no functional changes.

Nevertheless, our results must be interpreted with caution because a combined rehabilitation (conventional rehabilitation + EMG-commanded video games) was received for each participant. Future studies should be conducted comparing the effects of our experimental protocol versus conventional rehabilitation for UL in patients with MS.

Furthermore, it must be considered that these results cannot be generalized since a small sample size was recruited in this feasibility study. Note that this study is a case series, and the results might be influenced by factors such as the small sample size, the season when the intervention was performed (in summer or hot weather, MS patients get worse [40]), or the typical symptomatic variability of MS. However, it can be highlighted that no adverse effects were identified nor reported by participants, supporting the use of EMG video games on the UL functionality in patients with MS.

Another positive factor is the low scores obtained by SSQ and NASA-Task Load scales because it indicates that discomfort symptoms (dizziness, nausea, among others) and mental workload remained low, respectively. Visually induced effects or possible unpleasant sensations of virtual reality applications are a general concern. Hence, these favorable results regarding cybersickness are promising for the acceptance of using VR in MS treatment. While it is true that no immersive devices were used in this study, the developed video games are compatible with a VR headset. Thus, future work can compare the user experience using an either or not immersive setup, extending the target population.

Additionally, the experience using the EMG-controlled video games has been excellently rated by participants according to the satisfaction questionnaires. This aspect is very relevant in chronic pathologies such as MS, where the traditional treatments may seem repetitive and monotonous. Also, the utility and playability of the games have been highlighted by the users and clinicians. However, certain games have been difficult to perform, and the therapist's assistance was required. In most of the cases, the problem was that the gesture performed was different from the gesture calibrated. Thus, the therapist had to remember the patient to perform gestures as similar as possible to the ones calibrated.

On account of the above, one issue to be improved is the time spent in gesture calibration, since the time of treatment could be higher if the calibration procedure was faster. Currently, gesture calibration time takes around 5 minutes if all the gesture set is trained. However, the GUI can help the therapist to calibrate each gesture individually, allowing to fix only the gesture that is not properly recognized. Future work must consider additional methods, such as artificial intelligence or self-learning, in order to reduce the gesture calibration time and make the use of video games autonomously towards home treatment programs easy.

Regarding metrics acquired by the system, in addition to the games scores, it is possible to register automatically the muscle activation during the games' performance for further analysis. Literature highlights the potential of sEMG in prognosticating recovery, providing specific quantitative evidence for decision-making about treatments, and providing secondary information about the user's performance [39]. Therefore, an advantage of the proposed framework is the generation of richer information about the therapy session, and based on such information, other pathologies such as fatigue can be identified and measured. This requires further research and testing to validate this approach.

Finally, it can be noted that the current study has some limitations. Firstly, it lacks a control group, and the sample size was small, making it difficult to obtain statistical significance. Future studies should be conducted with more participants and a control group following a conventional rehabilitation program in order to compare the performance. Moreover, the obtained results cannot be generalized to all patients with MS, so these findings should be interpreted with caution. Further research and large-scale randomized controlled trials are essential before such novel rehabilitation techniques can be incorporated into clinical practice.

## 8. Conclusions

The results presented in this paper support the use of video games controlled by EMG sensors as a rehabilitative tool in patients with MS. In this study, an improvement in handgrip strength and gross manual dexterity was measured after a training protocol based on EMG-commanded video games. Although the number of patients is not sufficiently representative to give a statistical significance to the obtained results, it is nevertheless convincing about the effectiveness of the use of these games for a double function as a complementary rehabilitation instrument and an evaluation method to extract additional indicators about the user's performance. Treating patients with neurological damage in a practical and efficient way remains a challenge to achieve in terms of adherence. The development of this type of game aims to facilitate the administration of therapies to achieve better levels of adherence and therefore more intensive use and better outcomes.

Despite the positive results in this study, various issues must be solved in order to obtain full acceptance in clinical practice. A relevant aspect to improve is reducing the time spent in gesture calibration in order to increase the time available for therapy. For that purpose, the potential of the unity environment for including the gesture recognition system into the video game can be a promising research line towards simplifying the software tools required (Matlab would no longer be needed).

## Figures and Tables

**Table 1:** Sociodemographic data of participants.

	Age (years)	Evolution period (years)	EDSS score	Attendance (%)
Average	46.57	14.43	5.43	92.93
Median	49.00	17.00	6.00	100.00
SD	9.71	9.50	1.43	13.71
Minimum	29.00	1.00	3.00	62.50
Maximum	56.00	27.00	7.00	100.00

**Table 2:** Intervention: experimental protocol.

	Game and duration	Gesture sequence
Week 1 12 minutes	3 minutes per game	
	MYO-Gesture	Flexion/extension/pinch/grip/relax
	Arkanoid	Flexion/extension
	MYO-Space	Flexion/extension + grip
	MYO-Cooking	All gestures^†^

Week 2 12 minutes	3 minutes per game	
	MYO-Gesture	Flexion/extension/pinch/grip/extend
	Arkanoid	Pronation/supination
	MYO-Space	Flexion/extension + grip
	MYO-Cooking	All gestures^†^

Week 3 15 minutes	3′45″ minutes per game	
	MYO-Gesture	Flexion/extension/pinch/grip/relax
	Arkanoid	Pronation/supination
	MYO-Space	Flexion/extension + grip
	MYO-Cooking	All gestures^†^

Week 4 15 minutes	3′45″ minutes per game	
	MYO-Gesture	Flexion/extension/pinch/grip/extended
	Arkanoid	Flexion/extension
	MYO-Space	Flexion/extension + grip
	MYO-Cooking	All gestures^†^

Week 5 18 minutes	4′30″ minutes per game	
	MYO-Gesture	Flexion/extension/pinch/grip/extended
	Arkanoid	Pronation/supination
	MYO-Space	Pronation/supination + pinch
	MYO-Cooking	All gestures^†^

Week 6 18 minutes	4′30″ minutes per game	
	MYO-Gesture	Flexion/extension/pinch/grip/extended
	Arkanoid	Flexion/extension
	MYO-Space	Pronation/supination + pinch
	MYO-Cooking	All gestures^†^

Week 7 20 minutes	5 minutes per game	
	MYO-Gesture	Flexion/extension/pinch/grip/relax
	Arkanoid	Pronation/supination
	MYO-Space	Pronation/supination + pinch
	MYO-Cooking	All gestures^†^

Week 8 20 minutes	5 minutes per game	
	MYO-Gesture	Flexion/extension/pinch/grip/extended
	Arkanoid	Flexion/extension
	MYO-Space	Pronation/supination + pinch
	MYO-Cooking	All gestures^†^

^†^Flexion/extension/pronation/supination/pinch/grip/relax/extended.

**Table 3:** Jamar Handgrip dynamometer scoring in pounds (lb).

	More affected side			Less affected side		
	Baseline	Final	Follow-up	Baseline	Final	Follow-up
Participant 1	33	30	34	39	41	39
Participant 2	25	15	10	36	18	13
Participant 3	42	36	42	39	42	44
Participant 4	46	42	49	56	51	59
Participant 5	41	38	40	36	39	38
Participant 6	21	21	25	25	24	26
Participant 7	28	36	35	38	40	43
Median	33	36	35	38	40	39

**Table 4:** Box and Block Test scoring.

	More affected side			Less affected side		
	Baseline	Final	Follow-up	Baseline	Final	Follow-up
Participant 1	45	50	48	39	42	45
Participant 2	50	52	51	61	57	68
Participant 3	40	52	53	51	60	63
Participant 4	60	56	63	59	64	64
Participant 5	34	40	40	30	26	33
Participant 6	36	41	36	36	42	41
Participant 7	51	54	51	54	62	58
Median	45	52	51	51	57	58

**Table 5:** Nine Hole Peg Test scoring.

	More affected side			Less affected side		
	Baseline	Final	Follow-up	Baseline	Final	Follow-up
Participant 1	27.58	27.58	29.74	34.28	33.09	25.69
Participant 2	24.40	29.37	24.49	22.24	23.11	19.41
Participant 3	27.03	27.47	23.36	27.40	27.74	24.87
Participant 4	25.01	25.20	25.07	21.42	25.67	24.71
Participant 5	50.83	37.15	41.55	78.03	49.06	53.19
Participant 6	58.76	60.28	140.52	35.50	30.72	37.67
Participant 7	28.04	21.04	20.18	26.77	19.97	17.78
Median	28	28	25	27	28	25

**Table 6:** Statistical analysis results.

Outcome measure	Median (interquartile range)			*P* value (Friedman)	*P* value (Wilcoxon paired)		
	Baseline	Final	Follow-up		Bas.-fin.	Fin.-fol.	Bas.-fol.
Jamar MA ^†^	33 (17)	36 (17)	35 (17)	0.223			
Jamar LA ^‡^	38 (3)	40 (18)	39 (18)	0.396			
BBT MA	45 (15)	52 (13)	51 (13)	0.042^*∗*^	0,189	1,584	0,126
BBT LA	51 (23)	57 (20)	58 (23)	0.034^*∗*^	0,384	0,744	0,054
NHPT MA	27.58 (25.82)	27.58 (11.95)	25.07 (18.19)	0.717			
NHPT LA	27.40 (13.26)	27.74 (9.98)	24.87 (18.26)	0.368			
FSS	52 (11)	51 (16)	53 (14)	0.042^*∗*^	0,879	0,327	0,528
ABILHAND	40 (19)	40 (11)	41 (19)	0.582			
MSIS-29 physical	47.50 (60)	58.75 (22.50)	80 (47.50)	0.446			
MSIS-29 cognitive	38.88 (16.67)	52.77 (30.55)	44.44 (61.11)	0.540			

^†^MA: more affected side; ^‡^LA: less affected side; ^*∗*^significant.

**Table 7:** Results of the satisfaction questionnaires.

	Strongly disagree	Disagree	Neither agreement nor disagreement	Agree	Strongly agree
Q1	Accessibility (facilities)			3	4
Q2	Ease to use	1	3	3	
Q3	Fun games		4	3	
Q4	Graphic design and music in games	1	3	3	
Q5	Proper training protocol duration	1		4	2
Q6	Proper training session duration	2	1	3	1
Q7	Understanding of games mechanics		1	3	3
Q8	Aim-result of games		2	5	
Q9	Proper increasing difficulty in games		5	2	
Q10	Proper number of interactive sessions		3	4	
Q11	Duration of interactive sessions		4	2	1
Q12	Accessibility and intuitiveness	2	1	2	2
Q13	Attendance flexibility			3	4
Q14	Support of therapist			2	5
Q15	Clear instructions by therapist			2	5
Q16	Personalized service			2	5
Q17	Attendance at care center			2	5
Q18	Schedule flexibility			3	4
Q19	Objective scores in games		2	5	
Q20	Transferring gains to the ADL	2	3	2	
Q21	Expectations were satisfied	1	2	4	
Q22	Satisfaction level with protocol	1	1	5	

## Data Availability

Data are included in this paper.
